# β3-Adrenoreceptor Activity Limits Apigenin Efficacy in Ewing Sarcoma Cells: A Dual Approach to Prevent Cell Survival

**DOI:** 10.3390/ijms20092149

**Published:** 2019-04-30

**Authors:** Amada Pasha, Marina Vignoli, Angela Subbiani, Alessio Nocentini, Silvia Selleri, Paola Gratteri, Annalisa Dabraio, Tommaso Casini, Luca Filippi, Ilaria Fotzi, Claudio Favre, Maura Calvani

**Affiliations:** 1 Division of Pediatric Oncology/Hematology, Meyer University Children’s Hospital, 50139 Florence, Italy; amanda.pasha@yahoo.it (A.P.); marina.vignoli@unifi.it (M.V.); angela.subbiani@gmail.com (A.S.); annalisa.d.92@gmail.com (A.D.); tommaso.casini@meyer.it (T.C.); ilaria.fotzi@meyer.it (I.F.); claudio.favre@meyer.it (C.F.); 2 Department of Health Sciences, University of Florence, 50139 Florence, Italy; 3 NEUROFARBA Department, Pharmaceutical and nutraceutical section, University of Florence, 50019 Sesto Fiorentino, Italy; alessio.nocentini@unifi.it (A.N.); silvia.selleri@unifi.it (S.S.); 4 Laboratory of Molecular Modeling Cheminformatics & QSAR, NEUROFARBA Department, Pharmaceutical and nutraceutical section, University of Florence, 50019 Sesto Fiorentino, Italy; paola.gratteri@unifi.it; 5 Neonatal Intensive Care Unit, Medical Surgical Fetal-Neonatal Department, Meyer “University Children’s Hospital, 50139 Florence, Italy; luca.filippi@meyer.it

**Keywords:** apigenin, β3-adrenoreceptor, Ewing Sarcoma

## Abstract

Ewing Sarcoma (ES) is an aggressive paediatric tumour where oxidative stress and antioxidants play a central role in cancer therapy response. Inhibiting antioxidants expression, while at the same time elevating intracellular reactive oxygen species (ROS) levels, have been proposed as a valid strategy to overcome ES cancer progression. Flavonoid intake can affect free radical and nutritional status in children receiving cancer treatment, but it is not clear if it can arrest cancer progression. In particular, apigenin may enhance the effect of cytotoxic chemotherapy by inducing cell growth arrest, apoptosis, and by altering the redox state of the cells. Little is known about the use of apigenin in paediatric cancer. Recently, β3-adrenergic receptor (β3-AR) antagonism has been proposed as a possible strategy in cancer therapy for its ability to induce apoptosis by increasing intracellular levels of ROS. In this study we show that apigenin induces cell death in ES cells by modulating apoptosis, but not increasing ROS content. Since ES cells are susceptible to an increased oxidative stress to reduce cell viability, here we demonstrate that administration of β3-ARs antagonist, SR59230A, improves the apigenin effect on cell death, identifying β3-AR as a potential discriminating factor that could address the use of apigenin in ES.

## 1. Introduction

Ewing Sarcoma (ES) is one of the most common paediatric malignant tumours, accounting for 2% of all childhood cancers [1]. In recent decades, the therapeutic choice, consisting of a multi-drug chemotherapy regimen combined with radiotherapy and surgery, has significantly improved the survival to 70% in localised disease, but the outcome remains poor for patients with metastatic disease at diagnosis, occurring in approximately 25–30% of ES patients [2,3]. These cases often show resistance to multi chemotherapeutic agents [4]. It has been observed that response to the induction of cell death carried out by chemotherapeutic agents is different between ES patients and cell lines, reflecting the different levels of intracellular antioxidants and the variable ability to neutralize the action of reactive oxygen species (ROS) [5]. ES cells show a dysfunction in oxidative phosphorylation’s activity and a redox state imbalance due to their abnormal metabolic activity with an increased glucose uptake and activation of accelerated glycolysis that provide the energy required by cancer cells. These cells also show a high sensitivity to rapid changes in the intracellular redox environment. In this context a fine regulation of ROS production and detoxification is critical for the growth or reduction of an ES tumour. Therapeutic drugs act on the reduction of cellular antioxidant activity to promote oxidative stress increase and induction of cell death in ES. Cellular antioxidants are differentially expressed in ES cell lines and their levels can be associated with poor prognosis [6]. Glutathione (GSH) is one of the cellular antioxidants whose function is important in ES cell lines. It was observed that depletion of intracellular GSH decreases cell viability and enhances the efficacy of ROS-generating anticancer agents such as fenretinide [5].

Nutraceutical antioxidant supplement may improve tumour response to therapy and patient survival, leading to a long-term outcome or interference with chemo- and radiation- therapy by reducing their effectiveness [7,8]. Among nutraceutical antioxidants, flavonoid supplementation during cancer therapy is a controversial and questionable subject. Flavonoids have been implicated in tumour regression by either antioxidant or prooxidant activity. A plant flavonoid naturally abundant in vegetables and fruits is apigenin (5,7,4′-trihydroxyflavone) [9]. It is a bioactive flavonoid that shows anti-inflammatory, antioxidant, and anticancer properties against several human-cancer cell lines, including prostate carcinoma, colon carcinoma, breast cancer, leukemia cells, cervical carcinoma, lung cancer, and hepatoma [10,11,12,13,14,15,16,17,18,19]. The cellular effects of apigenin are associated with cell-cycle arrest and apoptosis induction mediated by the increase of p21 levels, regardless of the Rb status and p53 involvement [16,18,20,21], alteration of the Bax/Bcl-2 ratio, release of cytochrome C, and induction of Apaf-1, leading to caspase activation and PARP-cleavage [15,16,17,21,22].

Recently, β3 adrenergic receptors (β3-ARs) became incredibly attractive in cancer biology because of their role in reducing tumour growth and metastasis. Overexpression of β3-ARs has been associated with cancer growth, recruitment of circulating stromal cell precursors to the tumour sites, and enhancement of stem cell traits [23]. It has also been reported that β3-ARs stimulation exhibits dual antioxidant properties: it directly inhibits NADPHoxidase (NADPHox) activity, which regulates ROS production, and induces the expression of Catalase, which has a role as an endogenous antioxidant. Moreover, it has been observed that the stimulation of β3-ARs by noradrenaline increased the intracellular GSH levels [24].

In this study we investigated the effect of apigenin treatment on human Ewing Sarcoma cell lines, showing the partial decrease of cell viability and induction of cell apoptosis. Interestingly, the impact of apigenin in these cancer cells results in an improvement of the contemporaneous treatment with β3-ARs antagonist in a synergistic effect revealing a new possible approach for ES therapy.

## 2. Results

### 2.1. Apigenin Induces Cell Death and Inhibition of ROS and Antioxidant Activity in ES Cells

**Figure 1:**
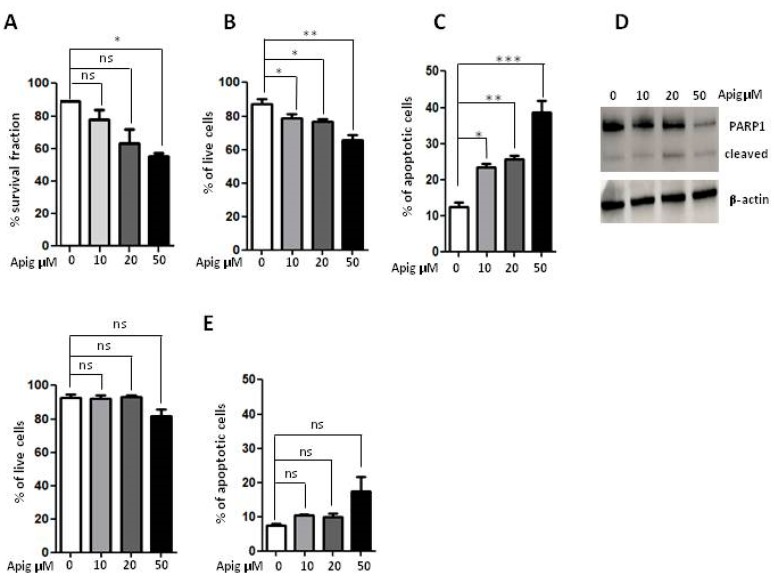
(**A**) Analysis of cellular viability with MTT survival experiment in human A673 ES cells after 24 h of treatment with apigenin (10-20-50 μM); (**B**) Percentage of live cells after Viobility^TM^ Fixable Dyes assay after 24 h of treatment with apigenin (10-20-50 μM); (**C**) The apoptotic effect of apigenin analysed after 24 h of treatment (10-20-50 μM); (**D**) Western Blot analysis of PARP1 enzyme after treatment with apigenin (10-20-50 μM) with β-actin as loading control; (**E**) Percentage of live cells after Viobility^TM^ Fixable Dyes assay and apoptotic effect on healthy lymphocytes after 24 h of treatment with apigenin (10-20-50 μM). Apig: apigenin, ns: not significant. *p* values for treatments: * *p* < 0.05, ** *p* < 0.01 and *** *p* < 0.001.

In order to investigate the effect of apigenin on the human ES cells, A673 cells were exposed to different concentrations of apigenin and the cellular viability was determined with four different methodologies. MTT analysis, Viobility^TM^ Fixable Dyes and Annexin V/PI staining showed that apigenin 50 μM decreased partially ES cell viability of about 35–39% (Figure 1A–C) and showed that the treatment affected early apoptosis to a higher extent than late apoptosis and necrocrotic cell death (Table 1 and Table 2). Moreover, investigation of the expression and cleavage of PARP-1 enzyme confirmed induction of the apoptotic process (Figure 1D). Furthermore, to exclude a massive toxic effect of high dose apigenin treatment, we performed cell viability assays on healthy human peripheral lymphocytes: no evidence of any toxic effect was observed after apigenin treatment (Figure 1E). Altogether, these results demonstrated that apigenin 50 μM reduced ES cell viability by activating the apoptotic pathway.

**Figure 2:**
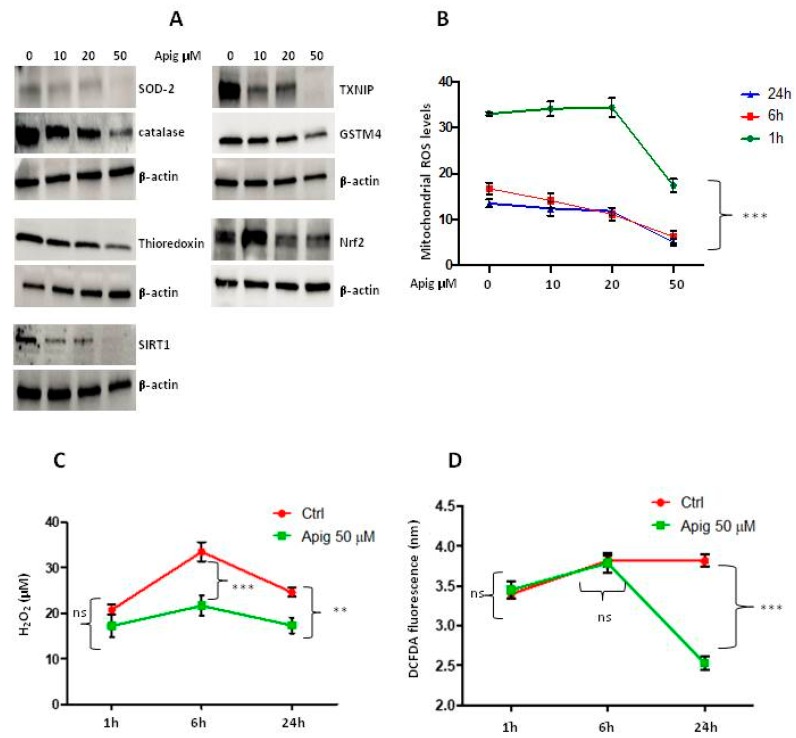
(**A**) WB analysis of apigenin effect on antioxidants expression: SOD2, Catalase, Thioredoxin, SIRT1, TXNIP, GSTM4, Nrf2 with β-actin as loading control; (**B**) Effect of apigenin after different times of treatment (1-6-24 h) on oxygen reactive species production (ROS) with MitoSOX^TM^ Red assay. (**C**) Effect of apigenin 50 μM after different times of treatment (1-6-24 h) on peroxide levels with Amplex^®^ Red Hydrogen Peroxidase Assay Kit (**D**) Effect of apigenin 50 μM after different times of treatment (1-6-24 h) on oxygen reactive species production (ROS) with DCFDA assay. ns: not significant. P values for treatments: ** *p* < 0.01, and *** *p* < 0.001.

The expression levels of antioxidants were examined upon treatment with apigenin at different concentrations 10-20-50 μM after 24 h. Apigenin treatment in A673 cells displayed a significantly lower amount of protein levels of all antioxidants examined, superoxide dismutase 2 (SOD2), Catalase, Thioredoxin, sirtuin-1 (SIRT1), thioredoxin interacting protein TXNIP (VDUP-1), glutathione S-transferase Mu4 (GSTM4), and nuclear factor erythroid 2-related factor 2 (Nrf2) (Figure 2A). The analysis of various ROS species levels measured at different times showed that the amount of most of ROS species decreased at the highest dose of apigenin (50 μM) after 24 h of treatment when it inhibits the expression of the most antioxidant proteins examined. Also, the amount of peroxide levels decreased after 6h of treatment (Figure 2B–D). Results indicated that apigenin partially reduced ES cell viability principally by inducing cell apoptosis.

### 2.2. Apigenin Controls ROS Levels by Activation of UCP2 and GSH Accumulation

**Figure 3:**
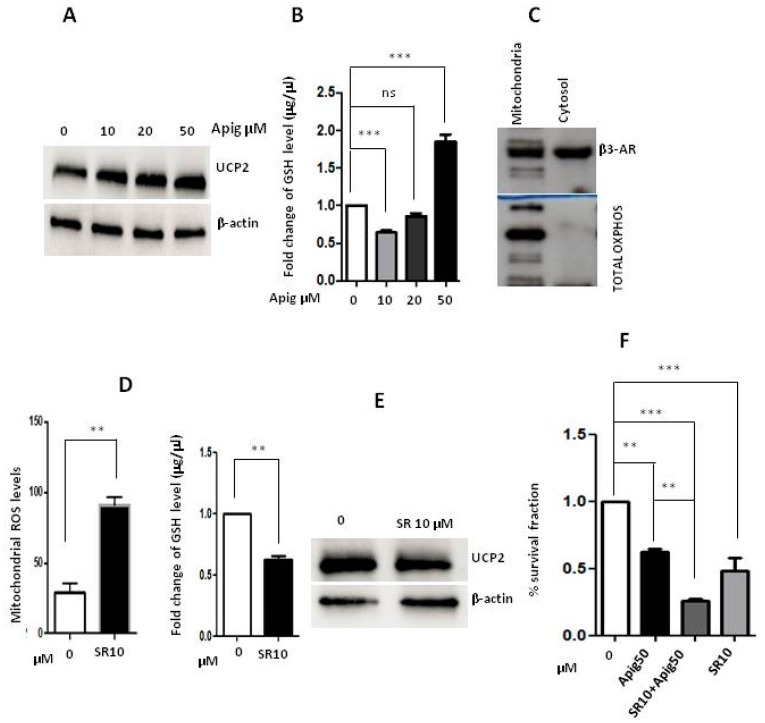
(**A**) Western Blot analysis of apigenin (10-20-50 μM) effect on UCP2 expression, with β-actin as loading control; (**B**) Measurement of reduced glutathione levels (GSH) after 24 h of treatment with apigenin; (**C**) WB analysis of β3-AR on mitochondria proteins; (**D**) Mitochondria mtROS measurement after treatment with β3-AR antagonist, SR59230A, at the concentration of 10 μM and measurement of GSH levels at the same time and concentration of SR59230A; (**E**) WB analysis of UCP2 expression after treatment with β3-AR antagonist SR59230A with β-actin as loading control; (**F**) MTT survival experiment with double treatment with SR59230A (10 μM) and apigenin (50 μM). SR10: SR59230A 10 μM, Apig50: apigenin 50 μM, ns: not significant. P values for treatments: ** *p* < 0.01 and *** *p* < 0.001.

Recently, it has been reported that a role of β3-adrenoreceptors in ROS balancing both in melanoma cells and in glioma cells, respectively controlling Uncoupling Protein 2 (UCP2) and glutathione levels [25]. In order to elucidate the mechanism by which apigenin reduced ROS levels, expression of UCP2 and GSH contents were analysed upon different time and doses of apigenin treatment. Results indicated that apigenin induced UCP2 protein expression and increased GSH levels after 24 h of treatment (Figure 3A,B), thus causing ROS levels decrease. Moreover, here we demonstrated the expression of β3-ARs in mitochondria of ES cells as it has been previously reported in melanoma cells [25] (Figure 3C). To address the involvement of β3-AR receptor in controlling ROS levels in ES cells, we used the selective antagonist of β3-AR, SR59230A. We showed an increase of mitochondrial ROS levels and an inhibition of GSH amount after 24h of treatment with SR59230A (Figure 3D). Interestingly, SR59230A inhibited the UCP2 expression in accord with previous data reported in melanoma cells (Figure 3E) [25]. These results indicate that the treatment with SR59230A could improve the effects of apigenin action by increasing ROS mitochondrial levels. Therefore, we tested the impact of the administration of apigenin and/or SR59230A (10 μM) on the survival of A673 cells (Figure 3F). Results clearly indicate that double treatment reduced cell viability with a higher extent respect to single treatments confirming the synergistic effect of both drug usage.

### 2.3. The Agonism of β3-AR Reproduces the Effect of Apigenin

**Figure 4:**
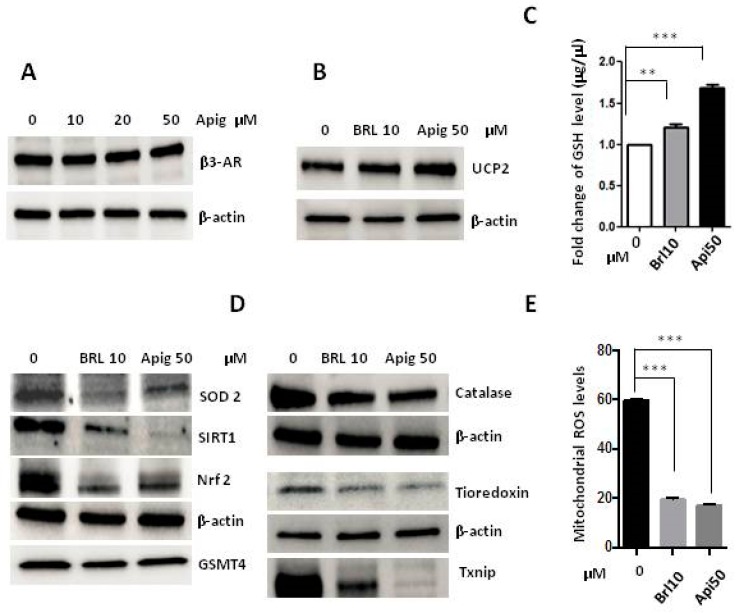
(**A**) Western Blot analysis of β3-AR expression after 24 h of treatment with apigenin (10-20-50 μM) with β-actin as loading control; (**B**) WB analysis of UCP2′s expression under treatment with β3-AR’s agonist, BRL37344 (10 μM) and apigenin (50 μM); (**C**)Measurement of GSH levels after 24 h of treatment with BRL37344 (10 μM) and apigenin (50 μM); (**D**) WB analysis of antioxidants SOD2, SIRT1, Nrf2, GSTM4, Catalase, Thioredoxin, TXNIP after 24 h of treatment with BRL37344 (10 μM) and apigenin (50 μM) with β-actin as loading control; (**E**) Mitochondrial mtROS measurement after treatment with BRL37344 (10 μM) and apigenin (50 μM) for 24 h. Brl10: BRL37344 10 μM, Apig50: apigenin 50 μM. *p* values for treatments: ** *p* < 0.01, and *** *p* < 0.001.

Even if β3-AR antagonism increased the levels of ROS, apigenin treatment did not increase β3-AR expression in A673 cells (Figure 4A), and so therefore we hypothesised that apigenin could work as β3-AR agonist. To address this question, we analysed the expression of UCP2 and the GSH production under the agonism of β3-AR with BRL37344 (10 μM), and we observed an increased expression of the protein and production of GSH comparable to the treatment with apigenin 50 μM (Figure 4B,C). Moreover, we observed that the expression of antioxidant levels was decreased after 24 h of treatment with BRL37344, and the same reduction was observed with apigenin treatment (Figure 4D). In addition, results clearly indicated that the agonism of β3-AR dramatically decreased ROS levels after 24 h of treatment in the same way as the apigenin treatment (Figure 4E).

### 2.4. Apigenin Could Be a β3-AR Agonist

To support the hypothesis on the β3-AR agonist profile of apigenin, in silico studies were performed and the ability of the ligand to bind the receptor was evaluated using two homology-built models (HM1 and HM2) based on the crystal structures of turkey β1-AR (pdb code 2Y03) [26] and human β2-AR (pdb code 3PDS) [27] as single template. The templates were chosen according to their sequence homology (55% and 42% for 2Y03 and 3PDS, respectively) as well as to their activation state. Indeed, agonist-bound templates were chosen basing on the putative agonist activity shown by apigenin in the biological assays. 

The conformational changes within the HMs induced upon ligand (apigenin) binding were evaluated by means of the induced fit docking procedure in order to allow both the receptor and the ligand to freely move during docking. The best protein-ligand complex (poses, one for each homology HM1 and HM2 models) resulting from this procedure were selected basing on the IFD scored values and binding energies estimated by applying the MM-GBSA method [28].

**Figure 5:**
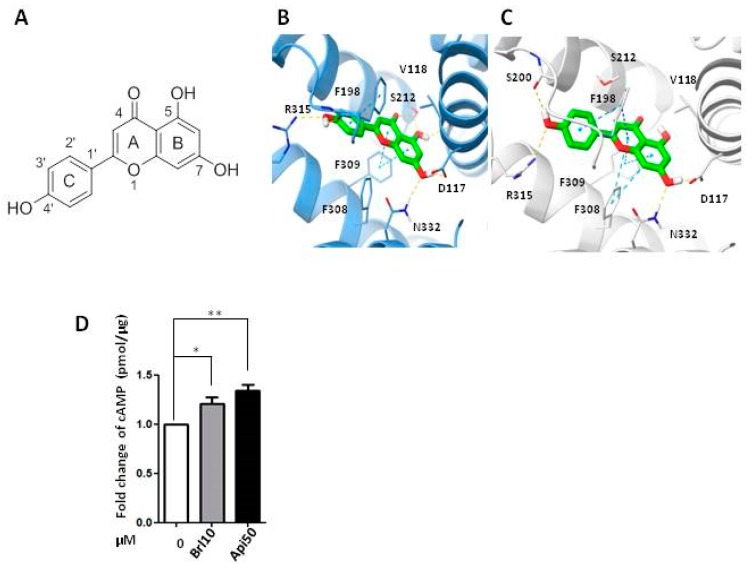
(**A**) Structure of apigenin. (**B**,**C**) Predicted binding mode of apigenin in the β3-AR binding pocket of (**B**) HM1 and (**C**) HM2; (**D**) Measurement of cAMP concentration after 30 min of treatment with BRL37344 (10 μM) and apigenin (50 μM). Brl10: BRL37344 10 μM, Apig50: apigenin 50 μM. *p* values for treatments: * *p* < 0.05 and ** *p* < 0.01.

Insights into the binding mode of apigenin with the two modelled targets revealed a key role played by the residues involved in the binding of β-ARs agonists/antagonists with the receptor, i.e., D117, F309, and N332. In particular, the 7-OH moiety of apigenin established two H-bond interactions with the side chains of D117 and N331, acting as a donor and acceptor, respectively. Moreover, the chromone core (i.e., the moiety formed by A+B in Figure 4A) of the ligand is sandwiched by F309 and V118, forming π-π and π-alkyl interactions. Other common interactions are a T-shaped π-π stacking engaged by the phenyl ring of apigenin (C in Figure 5A) and the side chain of F198 as well as a H-bond formed by the hydroxyl group in position 4′ and the guanidinium group of the R315 side chain. The outcomes of the in silico study substantiate the binding of apigenin to the β3-AR and support the findings of the biological assays.

As further proof, an analysis of the second messenger cyclic AMP (cAMP) levels was performed showing increased cAMP levels in the presence of BRL37344 and in the same way with apigenin 50 μM after 30 min of exposition, confirming that apigenin could act as β3-AR agonist (Figure 5B).

## 3. Discussion

The fine regulation of ROS production and detoxification is fundamental for the growth or reduction of a Ewing Sarcoma tumour. Therapeutic drugs that act on the reduction of cellular antioxidant activity, promote oxidative stress increase, and induction of cell death in ES. In this context, an antioxidant-inhibiting strategy has been evaluated in order to enhance the efficacy of some chemotherapeutics used in the standard treatment of ES such as doxorubicin and etoposide, which increase generation of ROS and oxidative stress in mitochondria [29]. In this study we demonstrated that apigenin induces partial cell death by activating the apoptotic pathway without increasing mitochondrial ROS production, which conversely is observed by administration of β3-AR antagonist.

Therapeutic strategies that are aimed to disrupt the redox homeostasis with bioactive nutrients of malignant cells of ES is constantly under debate. Unfortunately, despite the great interest, there is no consistent data regarding the use of apigenin and other flavonoids in humans as an anticancer therapy. Inconsistent reporting and study design for the investigation of flavonoids in both epidemiologic and intervention trials have significantly prevented development of clear recommendations about the intake of flavonoids to support or promote human health [30]. Apigenin has been reported to induce apoptosis in HepG2 cells by inhibiting Catalase activity and enhancing the expression of high levels of ROS, as well as preventing hepatocellular carcinogenesis via decreasing oxidative stress [31]. Flavonoids used in clinical practice may not confirm data of preclinical efficacy due to low/moderate anti-cancer activity when used alone at human physiological dosages [32,33,34]. Furthermore, usage at higher dosages has no known safety profile, with the possibility of unacceptable toxicities. Transition ions, as Cu2^+^ and Fe2^+^ present in biological systems, can affect the pro-oxidant activity of flavonoids, leading to inhibition of mitochondrial breathing. This activity of natural antioxidant plays an important role in their selective cytotoxicity toward cancer cells that contain more copper than normal cells [35,36,37,38].

Here we demonstrated that apigenin inhibits the expression of antioxidant proteins such as SOD2, Catalase, SIRT1, GSTM4, TXNIP, Thioredon1, and Nrf2, but increased the level of UCP2 and GSH which conversely are strongly inhibited by β3-AR antagonism. UCPs have been related to ROS production for the first time in 1997 in experiments where GDP, an inhibitor of UCP1, caused an increase of ROS production. Subsequent studies demonstrated that superoxide directly activates UCPs, leading to negative feedback controlling both ROS production and UCPs levels [39,40].

The β3-ARs antioxidant activity could be mediated by UCP2 protein expression that could work as a guardian for the redox homeostasis in ES cells. The redox homeostasis of cells is balanced by ROS generation and ROS quenching capacity. Undoubtedly, an imbalance in favor of increased ROS production within the cellular microenvironment by disruption of UCP2 signalling, and by inhibiting β3-ARs, can lead to excessive oxidative stress resulting in massive cell death.

The link between β3-ARs and UCP2 has been well described in white and brown adipocytes. Selective pharmacological β3-ARs stimulation has been shown to affect adipose tissue morphology and metabolism. The activity of CL-316,243, a potent and highly selective β3-ARs agonist [41] leads to improved thermogenesis in brown adipose tissue (BAT), lipolysis in white adipose tissue (WAT), and an acute decrease in food consumption [42,43]. Thermogenesis in BAT is mediated by activation by UCP1. β3-ARs agonists reduce fat stores, improved obesity-induced insulin resistance and increased brown adipocytes content in WAT tissue [44,45]. It is well known that β3-ARs control thermogenesis through activation of UCPs, in particular UCP1. UCPs maintain redox state of the cells in the respiratory chain transport. Interestingly, UCP2 has been shown to control GSH/GSSH in beta pancreatic cells [24,46].

More recently, data reported the β3-AR antioxidant activity by showing dual antioxidant properties: the reduction of NADPHox activity and induction of the expression of Catalase [46]. β3-ARs are expressed and functional in the human macrophages where their antioxidant effects lead to a potent anti-inflammatory response and play an important role in PPARγ activation through the Erk1/2 pathway [46]. In the second paper, authors show an increased intracellular concentration of GSH induced by GLCc protein in both U-251 MG cells and mouse astrocytes through noradrenaline-mediated β3-adrenoreceptor activation. This study revealed the importance of β3-ARs in the maintenance of GSH homeostasis in glioma cells by Gi/0-protein but not by Gs-protein in U-251 cells. These results indicated that the activation of intracellular signalling in response to β3-ARs stimulation may have been required for the induction of GSH by noradrenaline [24]. Even if apigenin inhibits antioxidant proteins, it works as β3-AR agonist, by avoiding the elevation of mitochondrial ROS useful to reach a massive cell death in ES cells.

In this work we confirm that the inhibition of antioxidants may be strategically useful in Ewing sarcoma therapy, and that the use of β3-ARs antagonist could be the limiting factor to reach a large amount of cell death.

## 4. Materials and Methods

### 4.1. Materials

Human Ewing Sarcoma (ES) cells A673 were purchased from the American Type Culture Collection (ATCC^®^ CRL-1598 ^TM^, Manassas, VA, USA). Dulbecco’s modified Eagle’s medium (DMEM) high-glucose, fetal bovine serum (FBS), penicillin-streptomycin, L-glutamine, and trypsin-enzyme were obtained from Euroclone Group (Pero, MI, Italy). Phosphate buffered saline (PBS) was purchased from Gibco (Gaithersburg, MD, USA). Dimethylsulfoxide (DMSO), apigenin (>97%), MTT (3-[4,5-dymethilthiazol-2-yl-]-2,5-diphenyltetrazolium bromide; thiazol blue) assay, BRL37344 and SR59230A were obtained from Sigma-Aldrich (St. Louis, MO, USA). Precast gels, PVDF membranes, Milk Blotting-Grade Blocker, and ECL, HRP Chemiluminescent Substrate Reagent kit were obtained from Biorad^®^ (Hercules, CA, USA); Tween^®^20 was obtained from Sigma-Aldrich. The primary antibodies used include the following: β3-adrenergic receptor (ab-77588), Catalase (ab-16731) and glutathione S-transferase Mu4 (GSTM4) from Abcam (Cambridge, MA, USA), superoxide dismutase-2 (SOD2) (sc-137254), β-actin (sc-1615), uncoupling protein 2 (UCP2) (sc-390189), erythroid 2-related factor 2 (Nrf2) (sc-305948) from Santa Cruz Biotechnology (Dallas, TX, USA), Thioredoxin 1 (MA5-14941) from Invitrogen by Thermo Fisher^®^ (Waltham, MA, USA); thioredoxin interacting protein TXNIP (VDUP-1) from Life Technologies (Waltham, MA, USA), Sirtuin 1 from Millipore (Darmstadt, Germany); Poly (ADP ribose) polymerase 1 (PARP-1) from Cell Signaling Technology (Beverly, MA, USA). The specific secondary antibodies, conjugated with horseradish peroxidase (HRP), anti-rabbit, anti-goat, and anti-mouse were purchased from Santa Cruz Biotechnology. Glutathione Fluorometric Assay Kit and cAMP Direct ImmunoAssay Kit (Colorimetric), were purchased from Biovision^®^ (Milpitas, CA, USA). Mitochondria isolation kit, and Annexin V-FITC kit were obtained from Miltenyi Biotec^®^ (Bergisch Gladbach, Germany). For ROS detection, MitoSOX^TM^ Red mitochondrial superoxide indicator from Invitrogen by Thermo Fisher^®^ was used. Viobility^TM^ Fixable Dyes were obtained from Miltenyi Biotec^®^. H2DCFDA Assay Kit and Amplex^®^ Red Hydrogen Peroxidase Assay Kit was obtained from Invitrogen by Thermo Fisher^®^. Separation Media Lymphosep for lymphocytes separation was obtained from Biowest (Nuaillè, France).

### 4.2. Cell Cultures

Human Ewing Sarcoma (ES) cells A673 were cultured in 100mm plates in DMEM high glucose medium supplemented with 10% fetal bovine serum (FBS), 1% of L-glutamine, 1% of penicillin-streptomycin and were maintained at 37 °C in a 5% CO_2_ humidified atmosphere incubator. Cells were usually stored in liquid nitrogen in a freezing solution, containing 95% complete DMEM medium and 5% DMSO and then plated in petri p100. For defrosting, the vials were rapidly brought to 37 °C by immersion in the thermostat bath, then centrifuged to remove the toxic DMSO from the cells, re-suspended in DMEM high glucose FBS 10, and appropriately plated.

Sub-confluent cells were detached from the plate with trypsin-enzyme after aspirating the medium and one wash with PBS to eliminate medium and serum residues. Then DMEM high glucose was added and the cell suspension obtained was counted and plated in fresh DMEM high glucose with appropriate dilutions. Human lymphocytes were isolated from peripheral blood with Separation Media Lymphosep to test the effect of apigenin in healthy control cells.

### 4.3. Cell Treatments

A673 ES cells were plated to reach 70% confluence in complete high-glucose DMEM medium. In order to promote cell entry into a G0 phase and better evaluate cells responsiveness to exogenous treatments, after 24 h the medium was removed, the cells were washed in PBS solution, and finally starved overnight with starvation medium (DMEM high glucose without FBS). The consequent morning, cells were treated with a single dose of apigenin at the concentrations of 10 μM, 20 μM, 50 μM [47,48,49,50] and subsequently left in an incubator for 24 h, then collected for the experiments. Apigenin was dissolved in DMSO 2.7 mg/mL to obtain a final concentration stock of 10mM, then appropriate dilutions from the stock solution were made for treatments.

### 4.4. MTT Assay

Viability of tumour cells after treatment with apigenin was detected by MTT (3-[4,5-dymethilthiazol-2-yl-]-2,5-diphenyltetrazolium bromide; thiazol blue) assay. A673 cells were transferred into a 96-well plate at a density of 10 × 10^4^ cells/well in 150 μL DMEM complete and were incubated with apigenin at different concentrations (10 μM, 20 μM, 50 μM) for 24 h. A total of 10μL of MTT was added to each well and incubated under darkness for 1h at 37 °C. Then, the culture medium was removed and 150 μL of DMSO was added to each well. The intensity of absorbance was detected at 570 nm using a spectrophotometer (PerkinElmer, Waltham, MA, USA).

### 4.5. Western Blot Analysis

After homogenization and protein quantification, samples (15–20 μg of total proteins) were loaded on SDS-PAGE and subjected to Western Blot analysis. Subsequently PVDF membranes were incubated for 1 h in slow agitation at room temperature in a blocking solution of non-fat dry milk 5% and Tween PBS 0.1% in order to avoid the formation of unspecific ties. Membranes were then incubated with the following primary antibodies: β3-adrenergic receptor, Catalase, Superoxide dismutase-2, TXNIP, Thioredoxin 1, Sirtuin 1, β-actin, UCP2, Nrf2, GSTM4. The primary antibody was added generally in a concentration of 1:1000 and incubated, in shaking, over-night at 4 °C. The next day membranes were washed three times with a washing solution containing Tween PBS 0.1% in order to remove unbound primary antibody in excess. Then, the specific secondary antibody, which was conjugated with horseradish peroxidase (HPR), was added, in a dilution of 1:5000 in Tween PBS 0.1% and incubated for 1 h. Chemiluminescent protein’s revelation was carried out with ECL reagent and developing of blots was carried out by Chemidoc Imaging System (Biorad^®^). To verify the application of equal amounts of protein, the intensity of the corresponding protein bands of interest was normalised based on that of the the β-actin band for each sample.

### 4.6. Glutathione Fluorometric Assay Kit

For the detection of reduced glutathione (GSH) the Glutathione Fluorometric Assay Kit was used, following the manufacturer instructions and intensity of fluorescence being analysed with spectrophotometer (PerkinElmer).

### 4.7. Cell Viability Analysis

For the detection and discrimination of live and dead cells (apoptotic, necrotic) in A673 line and lymphocytes, the Viobility^TM^ Fixable Dyes and Annexin V-FITC Kit were used after 24 h of treatment with apigenin at different concentrations, following the manufacturer’s instructions. Results were analysed by flow cytometry MACSQuant FACS (Miltenyi Biotec^®^).

### 4.8. cAMP Direct ImmunoAssay Kit

The Adenosine 3′,5′-cyclic monophosphate (cyclic AMP, cAMP) was measured by the cAMP Direct ImmunoAssay Kit (Colorimetric) after 30 min of treatment with BRL37344 (10 μM) and apigenin (50 μM) following the manufacturer’s instructions and absorbance at 450 nm detected using spectrophotometer (PerkinElmer).

### 4.9. ROS Analysis

For the intracellular ROS measurements, A673 cells plated into 24-wells plates at a density of 10 × 10^4^ cells in 1 mL complete high glucose DMEM medium, were treated with apigenin as described above. After 1-6-24 h of treatment, cells were stained with 1 μL of MitoSOX^TM^ Red mitochondrial superoxide indicator reagent at a concentration of 2.5 μM. After 15 min of incubation at room temperature under darkness, cells were washed with PBS, detached with 250 μL of trypsine-enzyme, spun at 1300 rpm for 5 min, and pellet was resuspended in 300 μL of PBS + 0.2% of FBS, and then evaluated by flow cytometry MACSQuant FACS (Miltenyi Biotec^®^). For peroxide measurements Amplex^®^ Red Hydrogen Peroxidase Assay Kit was used, following the manufacturer’s instructions. H2DCFDA Assay Kit was performed according to manufacturer’s instructions to measure levels of different species of ROS.

### 4.10. Mitochondria Isolation

To isolate the mitochondria from A673 cells the Mitochondria Isolation Kit was used. At the end of the kit procedure after the centrifugation at 13,000× *g* for 2 min at 4 °C, the supernatant was aspirated and the mitochondria pellet was resuspended in an adequate buffer for further analysis. We resuspended in lysis buffer for Western Blot analysis, and in PBS for analysis by flow cytometry MACSQuant FACS.

### 4.11. In Silico Methods

The primary sequence of the human β3-AR was retrieved from UniProt. The crystal structures of turkey β1-AR (2Y03) and human β2-AR (3PDS) were downloaded from the Protein Data Bank and used as a template in the homology modelling procedure. Prime module of the Schrödinger suite was used in the sequence alignment and model building procedures [51]. Then, the models were submitted to loop refinements and the quality checked by the analysis of the Ramachandran plots and evaluation of the QMEAN value.

The two HMs were prepared with Maestro (a of [51]) applying an energy minimization with RMSD value of 0.30 using the OPLS-3 force field. Apigenin structure was prepared by Maestro (a of [51]) and Macromodel (e of [51]) with the OPLS-3 force field was used for energy minimization.

The Induced Fit Docking protocol implemented in the Schrödinger package was used. The procedure consists of a Glide SP docking, (f of [51]) followed by a Prime refinement of the residue side chains within 5 Å and then by a final Glide XP docking of the ligand into the receptor in the refined conformation. The docking grid were centred on the centre of mass of the bound ligands. In the initial Glide SP docking, the vdW scaling was set to 0.7 for non-polar atoms of receptor and 0.5 those of the ligand.

The best poses for each model were submitted to MM-GBSA calculations (d of [51]) in VSGB solvent model enabling residue flexibility 5 A around the ligand to compute the binding free energies [52,53].

### 4.12. Statistical Analysis

In vitro data are presented as means ± Standard Deviation (SD) from at least three experiments. Results were normalised versus control expression levels.

Statistical analysis was performed using Graph Pad Prism software (GraphPad, San Diego, CA, USA) by one-way analysis of variance (ANOVA) and two-way analysis for all experiments except for the analysis of GSH levels after SR59230A treatment, followed by Bonferroni post hoc analysis.

## Figures and Tables

**Table 1:** Percentage of early apoptotic, late apoptotic and dead cells expressed by the annexin V assay in A673 cells and normal lymphocytes. APIG: apigenin.

A673 Cells	% Dead Cells			
	Early	Late	Necrotic	Total
0	2.59	4.95	5.36	12.09
APIG 10 μM	6.51	8.36	8.56	23.43
APIG 20 μM	7.89	9.70	8.11	25.70
APIG 50 μM	19.00	16.3	3.68	38.68
**Lymphocytes**				
0	4.26	1.35	2.02	7.63
APIG 10 μM	5.66	2.42	2.28	10.36
APIG 20 μM	5.74	2.23	2.05	10.02
APIG 50 μM	7.04	3.22	7.01	17.27

**Table 2:** Percentage of live cells expressed by the Viobility^TM^ Fixable Dyes assay in A673 cells and normal lymphocytes. APIG: apigenin.

A673 Cells	% Live Cells
0	87.20
APIG 10 μM	78.88
APIG 20 μM	76.90
APIG 50 μM	65.90
**Lymphocytes**	
0	92.70
APIG 10 μM	92.14
APIG 20 μM	93.41
APIG 50 μM	81.95
